# Sex and Strain Variation in Initial Sensitivity and Rapid Tolerance to Δ9–Tetrahydrocannabinol

**DOI:** 10.1089/can.2019.0047

**Published:** 2020-09-02

**Authors:** Cory Parks, Byron C. Jones, Bob M. Moore, Megan K. Mulligan

**Affiliations:** ^1^ Department of Genetics, Genomics and Informatics, College of Medicine, The University of Tennessee Health Science Center, Memphis, Tennessee.; ^2^ Department of Pharmaceutical Sciences, College of Medicine, The University of Tennessee Health Science Center, Memphis, Tennessee.

**Keywords:** C57BL/6, cannabinoid, DBA/2, sensitivity, THC, tolerance

## Abstract

**Background and Objectives:** For cannabis and other drugs of abuse, initial response and/or tolerance to drug effects can predict later dependence and problematic use. Our objective is to identify sex and genetic (strain) differences in initial response and rapid tolerance to Δ9–tetrahydrocannabinol (THC), the main psychoactive ingredient in cannabis, between highly genetically divergent inbred mouse strains—C57BL/6J (B6) and DBA/2J (D2).

**Experimental Approach:** Sex and strain responses relative to baseline were quantified following daily exposure (*i.p.*) to 10 mg/kg THC or vehicle (VEH) over the course of 5 days. Dependent measures included hypothermia (decreased body temperature) and ataxia (decreased spontaneous activity in the open field), and antinociception (increase in tail withdrawal latency to a thermal stimulus). Initial sensitivity to THC was defined as the difference in response between baseline and day 1. Rapid tolerance to THC was defined as the difference in response between days 1 and 2.

**Results:** B6 exhibited greater THC-induced motor activity suppression and initial sensitivity to ataxia relative to the D2 strain. Females demonstrated greater levels of THC-induced hypothermia and initial sensitivity relative to males. Higher levels of THC-induced antinociception and initial sensitivity were observed for D2 relative to B6. Rapid tolerance to THC was observed for hypothermia and antinociception. Much less tolerance was observed for THC-induced ataxia. D2 exhibited rapid tolerance to THC-induced hypothermia and antinociception at time points associated with peak THC initial response. Likewise, at the peak initial THC response time point, females demonstrated greater levels of rapid tolerance to hypothermic effects relative to males.

**Conclusions:** Both sex and genetic factors drive variation in initial response and rapid tolerance to the ataxic, antinociceptive, and hypothermic effects of THC. As these traits directly result from THC activation of the cannabinoid receptor 1, gene variants between B6 and D2 in cannabinoid signaling pathways are likely to mediate strain differences in response to THC.

## Introduction

Cannabis is used by millions of individuals both legally and illegally in the United States and worldwide. Estimates for individuals over the age of 12 from the United States in 2014 revealed that 2.5 million people used cannabis for the first time^1^ and that 22.2 million individuals used cannabis in the past month.^2^ Public attitudes and regulatory policies about the drug have also changed dramatically over time, resulting in increased use^3^ and a subsequent increased risk of abuse and use disorders.

Although estimates vary by year and epidemiological survey, lifetime prevalence of cannabis use disorder (CUD) may be as high as 8.3%.^4,5^ It is now clear that a significant number of individuals who use cannabis are at risk of developing CUD and associated withdrawal symptoms.^6–12^ CUDs are heritable and controlled by genetic factors^13,14^; however, only a handful of genes (i.e., *CADM2*, *NCAM1*, *NRG1*, *CSMD1*, and *CHRNA2*) have been associated with cannabis use or dependence in human genome-wide association studies.^15–19^ Thus, the genetic factors and biological processes underlying the transition between initial use and CUD remain largely unknown.

Pioneering work by Marc Schuckit demonstrated that individuals with lower initial sensitivity to the intoxicating effects of alcohol were at greater risk for later dependence and alcohol use disorders.^20^ Differences in initial response to a broad spectrum of drugs of abuse (opioids, stimulants, inhalants, and alcohol) often predict later dependence and substance use in humans^21^ or endophenoypes related to problematic use, such as tolerance and increased drug reward and self-administration in rodents.^22–25^ Similar associations between variation in initial subjective response to cannabis and later dependence are found for cannabis use in humans.^26^

Initial response to cannabis, THC, and other cannabinoid receptor 1 (CB1R) agonists has been measured across a range of doses among several inbred and outbred mouse strains (Table 1).^27–35^ However, few of these studies have directly compared strain differences in response and most do not include females, thus preventing analysis of sex differences. Despite marked variation among individuals in the acute response to cannabis in humans,^36^ little is known about the genetic factors mediating differential response or how initial sensitivity and tolerance may contribute to risk of CUD. Few studies systematically address possible genetic or sex differences in both initial response and tolerance to cannabinoids in human or animal models.

**Table 1.** Summary of Mouse Studies

Strain	Sex	Vendor	Response	Schedule	CB1R agonist	Dose	Tested phenotype	Post-inj. time	Sig.	Ref.
ddY^O^	M	Kuyodo, Saga, Japan	Initial Sensitivity	Acute	THC	10 mg/kg (*i.p.*)	Immobility (bar test)	60 min	^**^	^34^
ddY^O^	M	Kiwa Experimental Animal Laboratories, Wakayama, Japan	Initial Sensitivity	Acute	THC	6 mg/kg (*i.p.*)	Motor Depression (activity chamber)	60 min	^*^	^28^
							Hypothermia	180 min	^*^	
						10 mg/kg (*i.p.*)	Motor Depression (activity chamber)	60 min	^**^	
							Hypothermia	60 min	^**^	
							Hypothermia	180 min	^*^	
ICR^O^	M	Harlan Laboratories, Indianapolis, IN	Initial Sensitivity	Acute	Cannabis^†^	Dose–response (10, 50, 100, 200, and 300 mg, inhalation)	Motor Depression (activity chamber)	5 min	*n.s.*	^31^
							Antinociception (twl)	20 min	ED_50_=60 mg^*^	
							Immobility (ring test)	40 min	ED_50_=103 mg^*^	
							Hypothermia	60 min	*n.s.*	
			Initial sensitivity	Acute	THC	Dose–response (0, 0.5, 1, 2, 4, and 8 mg/kg, *i.v.*)	Motor Depression (activity chamber)	5 min	ED_50_=*NA*^‡^^*^	
							Antinociception (twl)	20 min	ED_50_=2.8 mg/kg^*^	
							Immobility (ring test)	40 min	ED_50_=1.9 mg/kg^*^	
							Hypothermia	60 min	ED_50_=3.4 mg/kg^*^	
Swiss Webster^O^	M	University of Arkansas for Medical Sciences Colony	Initial Sensitivity	Acute	THC	Dose–response (0,30, and 100 mg/30 L, inhalation)	Hypothermia	0 min	30 and 100 mg/30 L^*^	^33^
							Antinociception (twl)	0 min	100 mg/30 L^*^	
							Catalepsy (bar test)	0 min	*n.s.*	
							Motor Depression (activity chamber)	0 min	100 mg/30 L^*^	
					THC	Dose–response (0, 10, 30, and 100 mg/kg, *i.p.*)	Hypothermia	0 min	^*^	
							Antinociception (twl)	0 min	^*^	
							Catalepsy (bar test)	0 min	30 and 100 mg/kg^*^	
							Motor Depression (activity chamber)	0 min	^*^	
					JWH-018	(10, 30, and 100 mg/30 L, inhalation)	Hypothermia	0 min	10, 30, and 100 mg/30 L^*^	
							Antinociception (twl)	0 min	30 and 100 mg/30 L^*^	
							Catalepsy (bar test)	0 min	*n.s.*	
							Motor Depression (activity chamber)	0 min	10, 30, and 100 mg/30 L^*^	
					JWH-018	Dose–response (0, 1, 3, and 10 mg/kg, *i.p.*)	Hypothermia	0 min	^*^	
							Antinociception (twl)	0 min	^*^	
							Catalepsy (bar test)	0 min	3 and 10 mg/kg^*^	
							Motor Depression (activity chamber)	0 min	^*^	
					JWH-073	(10,30, and 100 mg/30 L, inhalation)	Hypothermia	0 min	30 and 100 mg/30 L^*^	
							Antinociception (twl)	0 min	100 mg/30 L^*^	
							Catalepsy (bar test)	0 min	*n.s.*	
							Motor Depression (activity chamber)	0 min	30 and 100 mg/30 L^*^	
					JWH-073	Dose–response (0, 3, 10, 30 mg/kg, *i.p.*)	Hypothermia	0 min	^*^	
							Antinociception (twl)	0 min	^*^	
							Catalepsy (bar test)	0 min	30 mg/kg^*^	
							Motor Depression (activity chamber)	0 min	^*^	
C57BL/6^I^	F	Jackson Laboratories, Bar Harbor, ME	Initial Sensitivity	Acute	THC	10 mg/kg (*i.p.*)	Motor Depression (activity chamber)	35 min	Y^*^; A^***^	^30^
	M					5 mg/kg (*i.p.*)			A^*^	
						10 mg/kg (*i.p.*)			A^**^	
ICR^O^	M	Charles River Laboratories, Wilmington, MA	Initial Sensitivity	Acute	THC	50 mg/kg (*i.p.)*	Catalepsy (ring test)	60 min	^*^	^35^
							Hypothermia	30 min	^*^	
							Motor Depression (activity chamber)	0 min	^*^	
							Antinociception (twl)	20 min	^*^	
C57BL/6^I^	M	Charles River Laboratories, Wilmington, MA	Initial Sensitivity	Acute	THC	50 mg/kg (*i.p.)*	Catalepsy (ring test)	60 min	^*^	
							Hypothermia	30 min	*n.s.*	
							Motor Depression (activity chamber)	0 min	^*^	
							Antinociception (twl)	20 min	*n.s.*	
DBA/2^I^	M	Charles River Laboratories, Wilmington, MA	Initial Sensitivity	Acute	THC	50 mg/kg (*i.p.)*	Catalepsy (ring test)	60 min	^*^	
							Hypothermia	30 min	^*^	
							Motor Depression (activity chamber)	0 min	^*^	
							Antinociception (twl)	20 min	^*^	
ICR^O^	M	Harlan Laboratories, Indianapolis, IN	Tolerance	Repeated	THC	0.5 day: 10 mg/kg (x1 daily *s.c*) + dose–response 24 h later (*i.v.*)	Antinociception	20 min	*n.s.*; ED_50_=1.10 mg/kg	^27^
							Motor Depression (activity chamber)	5 min	*n.s*.; ED_50_=0.61 mg/kg	
						1.5 day: 10 mg/kg (x2 daily *s.c.*) for 1 day +10 mg/kg (x1 daily *s.c*) + dose–response 24 h later (*i.v.*)	Antinociception	20 min	ED_50_=5.75 mg/kg^*^	
							Motor Depression (activity chamber)	5 min	ED_50_=4.16 mg/kg^*^	
						3.5 day: 10 mg/kg (x2 daily *s.c.*) for 3 days +10 mg/kg (x1 daily *s.c*) + dose–response 24 h later (*i.v.*)	Antinociception	20 min	ED_50_=8.76 mg/kg^*^	
							Motor Depression (activity chamber)	5 min	ED_50_=17 mg/kg^*^	
						6.5 day: 10 mg/kg (x2 daily *s.c.*) for 6 days +10 mg/kg (x1 daily *s.c*) + dose–response 24 h later (*i.v.*)	Antinociception	20 min	ED_50_=9.14 mg/kg^*^	
							Motor Depression (activity chamber)	5 min	ED_50_=8.09 mg/kg^*^	
C57BL/6J^I^	M	Jackson Laboratories, Bar Harbor, ME	Tolerance	Repeated	THC	2 days (10 mg/kg *i.p.* x1 every 72 h)	Motor Depression (open field)	0 min	J^*^; A^**^	^29^
DBA/2J^I^	M	Jackson Laboratories, Bar Harbor, ME	Tolerance	Repeated	THC	2 days (10 mg/kg *i.p.* x1 every 72 h)	Motor Depression (open field)	0 min	A^*^	
C57BL/6JArc^I^	M	Animal Resource Centre, Australia	Initial Sensitivity	Acute	THC	0.3, 1, 3 mg/kg (*i.p*.)	Catalepsy (bar test)	20 min	*n.s.*	^32^
							Hyperthermia	30 min	*n.s.*	
							Antinociception (twl)	45 min	*n.s.*	
							Motor Depression (open field)	30 min	*n.s.*	
						10 mg/kg (*i.p*.)	Catalepsy (bar test)	20 min	^*^	
							Hypothermia	30 min	^*^	
							Antinociception (twl)	45 min	*n.s.*	
							Motor Depression (open field)	30 min	^*^	
			Tolerance	Repeated	THC	3, 5, 7, 12 or 14 consecutive treatment days at 0.3, 1, 3, or 10 mg/kg (*i.p*.) per day	Catalepsy (bar test)	20 min	*n.s.*	
							Hypothermia	30 min	*n.s.*	
						14 consecutive treatment days at 0.3, 1, or 3 mg/kg (*i.p*.) per day	Antinociception (twl)	45 min	*n.s.*	
						14 consecutive treatment days at 10 mg/kg (*i.p*.) per day	Antinociception (twl)	45 min	^***^	
						15 and 21 consecutive treatment days at 0.3, 1, or 3 mg/kg (*i.p*.) per day	Motor Depression (open field)	30 min	Day 21; 1 mg/kg^*^ and 3 mg/kg^**^	
						15 and 21 consecutive treatment days at 10 mg/kg (*i.p*.) per day	Motor Depression (open field)	30 min	Day 15^**^ and Day 21^***^	

Post-inj. Time=Time point trait was measured relative to CB1R agonist exposure. Sig.=Significance level; *p*<0.05^*^, *p*<0.01^**^, *p*<0.001^***^. Ref.=Reference cited. twl=tail withdrawal latency in response to a thermal stimulus. J=juvenile (32 days old) and A=adult (73 days old). n.s.=not significant.

^†^ Cannabis contained 3.46% THC, 0.18% cannabinol, 0.17% cannabidiol, 0.14% cannabigerol, and 0.05% tetrahydrocannabivarin.

^I^ Inbred strain.

^O^ outbred strain.

^‡^ THC significantly inhibited locomotor activity; EC50 was not calculated because an effect >50% was not observed at the highest dose.

In this study, we begin to address these gaps in knowledge by profiling the response to the main psychoactive substance in cannabis, THC, in males and females from highly genetically divergent C57BL/6J (B6) and DBA/2J (D2) inbred strains of mice. These strains differ at millions of loci^37^ and are the progenitor strains for a large and well-characterized panel of recombinant inbred strains, the BXD population.^38^ Many phenotypes show heritable (controlled by genetic factors) differences among the B6 and D2 parental strains and their recombinant inbred BXD progeny, including response to drugs of abuse—ethanol, nicotine, methamphetamine, cocaine, and opioids.^39–43^ The gene variants underlying these and other heritable differences between B6 and D2 can be identified using forward genetic mapping approaches in the BXD panel.^44^

To begin to address potential genetic variation in specific molecular signaling pathways that mediate response to THC, we measured behavioral and physiological responses directly related to THC activation of the CB1R in male and female B6 and D2 mice. These responses include diminished locomotor activity, hypothermia, and longer latencies to respond to a thermal stimulus.^45–49^ Specifically, we quantified initial sensitivity as the acute response upon first exposure to THC and rapid tolerance as the response upon the second exposure to THC.

Rapid tolerance was included because repeated daily administration of THC in humans and animals produces rapidly diminished physiological responses (rapid tolerance) within just a few days. This response may be linked to the rapid adaptation or desensitization of the main neuronal receptor of the endocannabinoid system, CB1R.^50^ We also compared overall rates of tolerance/desensitization following five consecutive daily exposures to THC.

In this study, we provide the first report of sex differences in the hypothermic response to THC and genetic (strain) differences in initial sensitivity and rapid tolerance to the ataxic and/or analgesic effects of THC. Identification of genetic differences influencing sensitivity and tolerance to THC is important for understanding negative aspects associated with acute (e.g., motor depression and intoxicating effects) and chronic use (e.g., cannabinoid dependence and withdrawal), and for developing the therapeutic potential (e.g., analgesic effects) of the endocannabinoid system.

## Materials and Methods

### Animals

B6 and D2 mice (at least 20 per strain) were purchased from the Jackson Laboratory or bred in-house as described below. All mice were at least 60 days of age before testing. At least 1 week before testing, animals were separated into individual housing and handled daily. To reduce stress and anxiety responses throughout the study, handling (with the exception of *i.p.* injections) consisted of lifting mice in either cupped hands (avoiding any lifting by the tail) or a plastic lid from a pipette tip box.^51–53^ Food and water were provided *ad libitum* and mice were maintained on a 12-h light:12-h dark cycle with lights on at 0600 h. All testing was performed during the light cycle from 0700 h to 1600 h. All animal activities were approved by the University of Tennessee Health Science Center Institutional Animal Care and Use Committee.

### THC and vehicle formulation

THC was formulated in an ethanol:cremophor:saline (5:5:90) vehicle (VEH) followed by filter sterilization. The resulting formulation was stored in the dark and under (4°C) refrigeration in a septum-sealed vial. A new formulation was generated for each cohort of mice and used within 1 week of formulation. The VEH was prepared in the same manner. THC and VEH were administered by intraperitoneal (*i.p.*) injection at a dose of 10 mg/kg, such that a 30 g mouse received a 100 μL injection. The 10 mg/kg dose was selected based on previous pre-clinical studies that demonstrate significant responses (ataxia, hypothermia, and/or antinociception) in a number of strains at this dose (Table 1).

### THC formulation stability

Five microliters of the formulation was removed immediately after preparation and 8 days post-preparation, and each diluted with 100 μL of methanol. The samples were analyzed immediately following sample preparation using high-resolution mass spectroscopy (HRMS) on a Waters Xevo G2+S QTOF (Milford, MA) system in negative mode.

A reversed-phase BEH C18 analytical column (2.1×50 mm, 1.7 mm particle size; Waters) was used for the LC separation. The mobile phase was 95% water with 5% acetonitrile (solution A) and acetonitrile (solution B). The extracts were resolved using a 5-min gradient elution of 20% solution B; 1.5–3.0 min of 20% to 100% solution B; 3.0–4 min of 100% solution B; and 4–4.5 min of 100% to 20% solution B at a flow rate of 0.3 mL/min.

A PDA detector (Waters, Milford, MA) was used to measure the UV chromatogram before mass spectrometer analysis. The THC eluted at 3.75 min with a molecular mass of 313.2017 [M-H] (calculated for m/z C21H29O2 [M-H] 313.2173). The THC peaks were integrated and the formulation stored for 8 days was found to contain 95.2% of the initial THC used in the formulation.

### Phenotyping pipeline A

An initial pilot study was performed first in B6 and D2 male mice (20 per strain) purchased from the Jackson Laboratory. A separate cohort of female mice (18 per strain), also purchased from the Jackson Laboratory, was tested ∼1 year later. Mice were randomly assigned to the THC or VEH groups. The number of mice tested in this pipeline was as follows: 4 VEH B6 females, 4 VEH D2 females, 4 VEH B6 males, 4 VEH D2 males, 14 THC B6 females, 14 THC D2 females, 16 THC B6 males, and 16 THC D2 males.

**FIG. 1.**
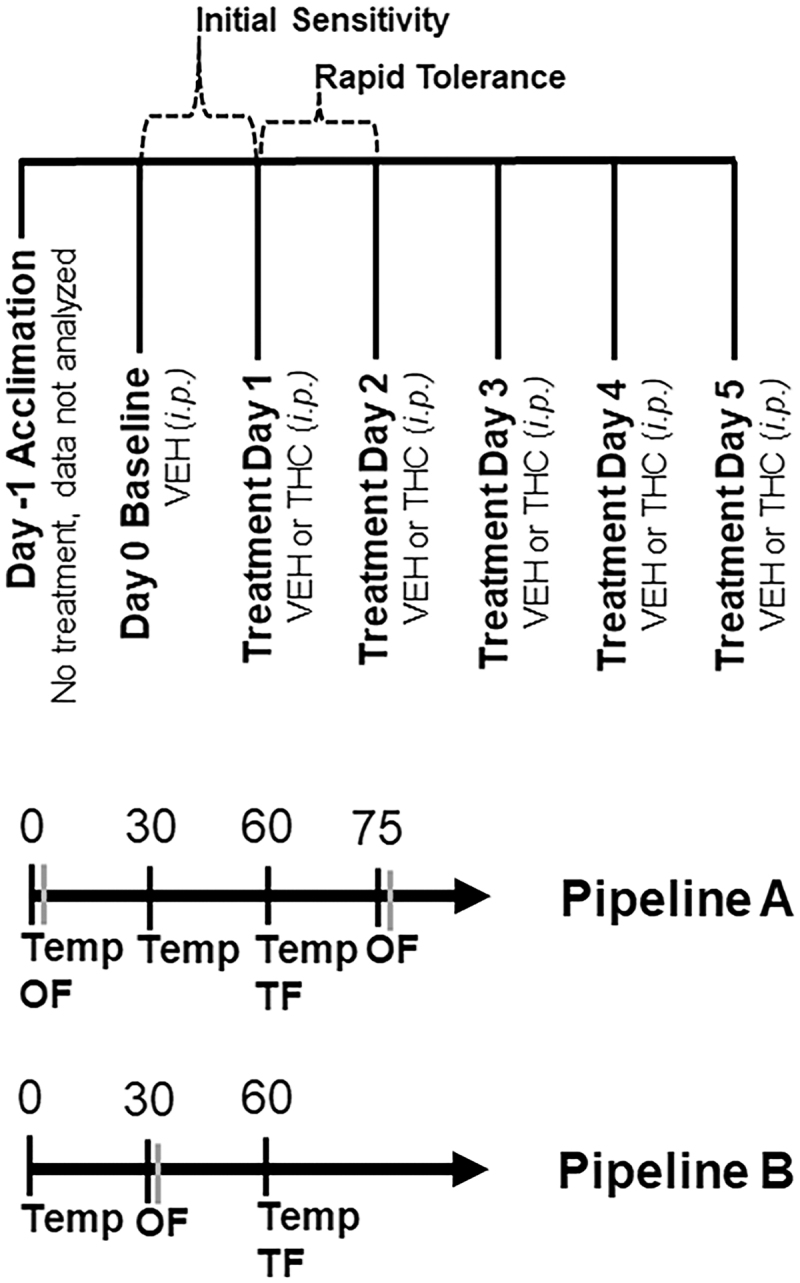
Overview of Experimental Pipelines. An overview of treatments and test days can be found in the top panel. Testing occurred over 7 days. On the first day, mice were acclimated to the testing paradigm for testing pipelines A and B. Animals were not treated and data collected on this day were not analyzed. Baseline data were collected on day 0 and all mice received a VEH injection (100 μL per 30 g body weight, *i.p.*). On days 1 through 5, mice received either VEH or THC (10 mg/kg, *i.p.*) treatment depending on their random assignment to either condition. At the indicated time post-injection of VEH or THC, and on each day, mice were tested for the effect of treatment on hypothermia (body temperature or Temp), ataxia (spontaneous motor activity over 10 min in the open field or OF), and analgesia/antinociception (tail withdrawal latency to a thermal stimulus or TF). VEH, vehicle; THC, Δ9–tetrahydrocannabinol.

Mice were tested over 7 consecutive days (Fig. 1). No injections were given on day −1 (acclimation). On day 0 (baseline), all subjects received a VEH injection (100 μL per 30 g, *i.p.*), and on days 1 through 5 (treatment), all subjects received a VEH or THC injection (*i.p.*). On each day, body temperature and motor activity were measured at multiple time points (Fig. 1). Because repeated testing within session can influence behavior, tail withdrawal latency in response to a thermal stimulus was measured at a single time point.

Time points and THC dose (10 mg/kg) were selected based on literature survey (Table 1). Previous studies demonstrated that 10 mg/kg (*i.p.*) THC is the lowest dose that reliably produced significant effects in ataxia, hypothermia, and/or antinociception relative to controls across a range of mouse strains (predominately male) (Table 1).

Temperature was measured using a ThermoWorks digital thermometer with rectal probe adaptor at time 0, 30, and 60 min post-injection. Analgesia was measured 60 min post-injection as the tail withdrawal latency to a thermal stimulus using a tail flick assay. In this assay, mice were gently restrained in a 50 mL conical tube or by hand, and the tail was submerged ∼2 cm in a 52°C water bath. The latency to remove the tail in response to the thermal stimulus was recorded to the nearest 1/10 sec. Average tail flick latency was recorded based on two independent observers.

Motor activity was measured as the time spent mobile (spontaneous motor activity) in a 40×40 cm open field at 0 and 75 min post-injection. Mice were gently transported by hand or in a clear plastic box to the center of the open field and activity was video recorded for 10 min. Immobility was detected using AnyMaze (Stoelting) software and was operationally defined as no movement for at least 3 sec. Mice were habituated to the test room for 1 h before testing.

### Phenotyping pipeline B

Male and female mice from a breeding colony at UTHSC (less than six generations from the Jackson Laboratory stock) were used. Mice were randomly assigned to THC or VEH groups. Males and females of both strains were tested together in several small batches. Strain and sex were counterbalanced across batches and within daily sessions. Up to eight mice were included in each session and up to four sessions were run per testing batch. Mice were returned to their home cage between tests. Mice were habituated to the test room for 1 h before testing.

The testing schedule was similar to pipeline A, with the exception that the schedule was streamlined to increase throughput by measuring phenotypes at a single time point (Fig. 1) over a shortened time period (60 min), relative to pipeline A (85 min). Mobility in the open field was measured at 30 min post-injection and core body temperature and analgesia were measured at 60 min post-injection. The number of mice tested in this pipeline was as follows: 6 VEH B6 females, 6 VEH D2 females, 12 VEH B6 males, 14 VEH D2 males, 16 THC B6 females, 13 THC D2 females, 17 THC B6 males, and 17 THC D2 males.

### Statistical analysis

Data from all cohorts and both phenotyping pipelines were combined for statistical analysis. Statistical outliers (greater than 2 standard deviations from the mean for each sex and strain by day and trait) were excluded from further analysis as were individuals with technical artifacts (e.g., bad injection, AnyMaze software failure, temperature probe failure, or mouse with obvious health issues). Data from days 0 through 5 were included in the analysis. Raw trait data (mobility and tail flick latency) for each individual were transformed relative to baseline (day 0) by subtracting trait values for each day by the trait values for day 0. Raw temperature trait data for each individual were transformed relative to baseline (time 0) by subtracting trait values for each day (time 30 or 60) by trait values for time 0.

For each individual, response to treatment (THC or VEH) was calculated as the area under the curve (AUC) using the plyr and MESS packages in R. The spline function [type=c(“spline”)] and the absolute area (negative and positive areas combined; absolute area=TRUE) were used with MESS package defaults for the auc function to calculate AUC.

Omnibus ANOVAs were then performed on the AUC data using base functions in R to determine trait response based on a number of independent factors. Time mobile in the open field (a measure of the motor depressant effect of treatment) was evaluated using a multifactor ANOVA: AUC_mobile_ ∼ Treatment*Strain*Time*Sex. Body temperature (a measure of the hypothermic effect of treatment) was evaluated using a multifactor ANOVA: AUC_temperature_ ∼ Treatment* Time* Sex*Strain. Tail flick latency to a thermal stimulus (a measure of the antinociceptive effect of treatment) was evaluated using a multifactor ANOVA: AUC_temperature_ ∼ Treatment*Strain*Sex. Factor order was arranged to maximize impact (F-value) of each factor on trait variation.

*Post-hoc* analysis [multifactor ANOVA and Tukey's honest significant difference (HSD)] was then performed to specifically evaluate the effect of sex and strain on initial sensitivity and rapid desensitization to THC as described below.

Initial sensitivity was calculated for the THC group as the difference between day 1 (acute THC treatment) and day 0 (baseline). This difference score was calculated for each individual and then averaged by strain and sex. Difference scores provide a more direct method to investigate drug response and are more robust to the effects of batch.^54^ To determine the effect of strain and sex on initial sensitivity to THC for each trait (y), multifactor ANOVA in the form of y ∼ Strain*Sex was performed using base functions in R. *Post-hoc* analysis to determine pairwise significance between strains for each sex and time point was performed using Tukey's HSD test (TukeyHSD base function in R).

Rapid tolerance was calculated for the THC group as the difference between day 2 (second THC exposure) and day 1 (acute THC treatment). This difference score was calculated for each individual and then averaged by strain and sex. To determine the effect of strain and sex on rapid tolerance to THC for each trait (y), multifactor ANOVA in the form of y ∼ Strain*Sex was performed using base functions in R. *Post-hoc* analysis to determine pairwise significance between strains for each sex and time point was performed using Tukey's HSD test as described above.

## Results

### Sex and strain differences in psychomotor response to THC

**FIG. 2.**
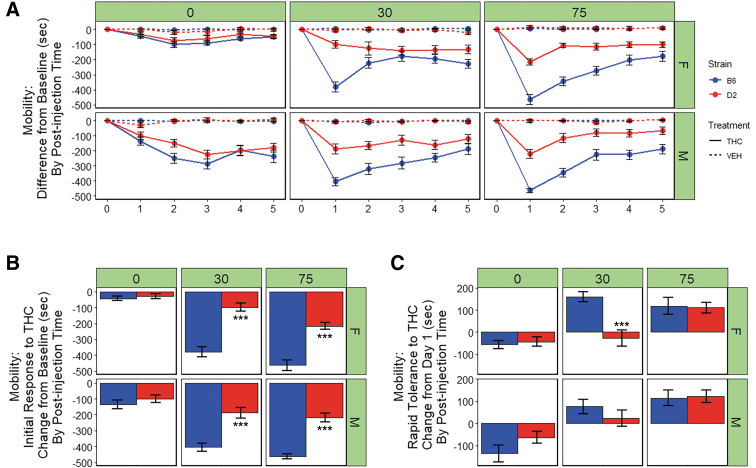
Motor response to THC. **(A)** Time mobile in seconds (sec) in the open field is shown for B6 and D2 females (top panel) and males (bottom panel) at three time points (0, 30, or 75 min) post-injection of THC (10 mg/kg) or VEH. Trait data for every individual have been transformed relative to baseline (day 0) by subtracting trait values for each day by the trait values for day 0 and averaging data by strain, sex, treatment, day, and post-injection time. Data represented as mean±SEM. Ataxia is evident as early as 0–10 min (0-min time point) post-injection on day 1 and is more prominent in males. Peak initial response to THC occurs at 75 min post-injection on day 1. Both males and females of the B6 strain are more sensitive to the ataxic effects of THC relative to both sexes of the D2 strain, and males are more sensitive than females. Rapid tolerance is evident upon second exposure to THC and results in a modest ∼25% (B6) to ∼50% (D2) increase in motor activity. However, full desensitization (return to baseline levels) is not observed after five consecutive treatments. **(B)** Initial response to THC is shown as the difference between day 1 and 0 (baseline) for the THC treatment group (average of each individual's difference score, VEH group not shown). Significant strain differences in initial sensitivity to the ataxic effects of THC were evident on day 1 for both males and females at the 30 and 75 min post-injection time points based on Tukey's HSD test. **(C)** Rapid tolerance to THC is shown as the difference between day 2 and 1 (THC treatment group only). Rapid tolerance to the ataxic effects of THC is not evident at 0 min post-injection. Relative to D2 mice, B6 mice demonstrate significantly greater rapid tolerance to the ataxic effects of THC at 30 min post-injection. B6 females exhibited significantly greater rapid tolerance relative to D2 females at 30 min post-injection. Significance defined as *p*<0.05*, *p*<0.01**, and *p*<0.001***.

Results are summarized in Figure 2A. Compared to VEH, treatment with THC (10 mg/kg, *i.p.*) resulted in a significant reduction in the time spent mobile in the open field [F(1,284)=353.4, *p*<0.001]. There was also a significant main effect of strain [B6 > D2; F(1,284)=88.2, *p*<0.001], post-injection time [75 min >30 min >0 min; F(1,284)=43.6, *p*<0.001], and sex [M > F; F(1,284)=20.8, *p*<0.001] on the reduction of locomotor activity.

The B6 strain was more sensitive to the motor depressant effects of THC relative to D2 and this difference was enhanced at 30 and 75 min post-injection as indicated by significant interactions between treatment and strain [F(1,284)=43.3, *p*<0.001], strain and post-injection time [F(1,284)=32.7, *p*<0.001], and treatment, strain, and time [F(1,284)=8, *p*<0.01]. In addition, there were significant interactions between treatment and sex [F(1,284)=10.0, *p*<0.01], time and sex [F(1,284)=29.6, *p*<0.001], and treatment, time, and sex [F(1,284)=29.6, *p*<0.001], with males exhibiting an earlier (post-injection time 0) and more pronounced response to the motor effects of THC relative to females and VEH treatment at 30 min post-injection.

Significant strain differences in initial sensitivity to the ataxic effects of THC upon first exposure were observed at both the 30-min [B6 > D2; F(1,48)=72.7, *p*<0.001)] and the 75-min [B6 > D2; F(1,78)=92.6, *p*<0.001)] time point (Fig. 2B). At both 30 min post-injection of THC, B6 males and females demonstrated greater initial sensitivity to the locomotor depressing effects of THC relative to D2 males and females (*p* adjusted <0.001; Fig. 2B). No significant main effects of sex or interaction effects were observed at these time points. However, a trend for greater initial sensitivity to the ataxic effects of THC in males relative to females was observed at the 30-min time point [F(1,48)=72.7, *p*=0.06)]. A significant main effect of sex on initial sensitivity to the ataxic effects of THC upon first exposure was observed at 0 min [M > F; F(1,78)=14.3, *p*<0.001] post-injection of THC (Fig. 2B). No significant effect of strain or interaction effects was observed at this early time point.

Rapid tolerance to the ataxic effects of THC following two consecutive treatments (day 2) was evident in males and females of both strains at 30 and 75 min post-injection, but not at 0 min post-injection(Fig. 2A). A significant main effect of strain [B6 > D2; F(1,48)=14.6, *p*<0.001)] and a significant sex-by-strain interaction effect [F(1,48)=4.2, *p*<0.05)] on rapid tolerance upon second exposure to THC were observed at 30 min post-injection only (Fig. 2C). *Post-hoc* analysis of the 30 min post-injection time point revealed that B6 females exhibited significantly greater rapid tolerance to the ataxic effects of THC relative to D2 females (*p* adjusted <0.001, Fig. 2C).

For B6 males and females, rapid tolerance to the ataxic effects of THC upon second exposure accounted for an ∼25% change in the direction of baseline levels of activity at the time of maximal initial response (THC group, 75 min post-injection, Fig. 2A). For D2 males and females, rapid tolerance to the ataxic effects of THC upon second exposure accounted for an ∼50% change in the direction of baseline levels of activity at the time of maximal initial response (THC group, 75 min post-injection, Fig. 2A). For both strains and sexes, and at every time point post-injection, the return to baseline was still incomplete after five consecutive daily exposures to THC (Fig. 2A).

### Sex and strain differences in the hypothermic response to THC

**FIG. 3.**
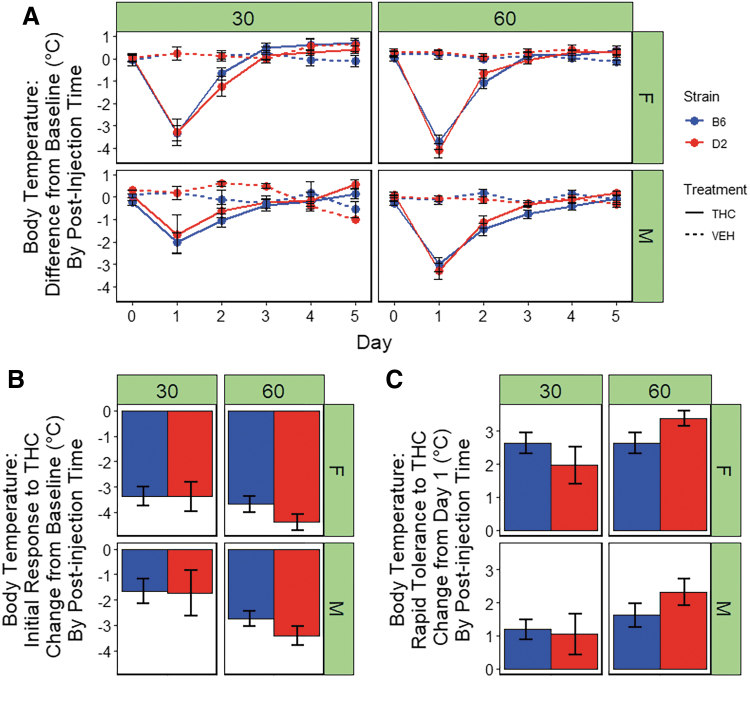
Hypothermic response to THC. **(A)** Body temperature (°C) is shown for B6 and D2 females (top panel) and males (bottom panel) at three time points (0, 30, or 60 min) post-injection of THC (10 mg/kg) or VEH. Trait data for every individual have been transformed relative to baseline (time 0) by subtracting 30- and 60-min trait values for each day by the trait values for time 0 and averaging data by strain, sex, treatment, day, and post-injection time. Data represented as mean±SEM. Profound hyperthermia is evident in both strains at 30 min post-injection on day 1 and is more pronounced in females. Peak initial response to THC occurs at 60 min post-injection. Rapid tolerance upon second exposure results in a substantial increase in body temperature toward baseline in both strains and sexes. Desensitization is complete in both strains and sexed by the third exposure to THC. **(B)** Initial response to THC is shown as the difference between day 1 and 0 (baseline) for the THC treatment group. Females demonstrate significantly greater hypothermia after a single exposure to THC at 30 and 60 min post-injection relative to males. At the 60-min time point only, a significant main effect of strain was observed, in which the D2 strain exhibits greater sensitivity to the hypothermic effects of THC relative to the B6 strain. There were no significant interaction effects observed between strain and sex. **(C)** Rapid desensitization to THC is shown as the difference between day 2 and 1 (THC treatment group only). Desensitization to the hypothermic effects of THC is evident for both strains, sexes, and post-injection times by the third day of treatment. Females demonstrate significantly greater rapid tolerance to the hypothermic effects of THC at all time points. At the 60-min time point, the D2 strain exhibits slightly greater rapid tolerance relative to B6.

There were significant main effects of treatment [THC > VEH; F(1,181)=106.4, *p*<0.001], post-injection time [60 min >30 min; F(1,181)=11.6, *p*<0.001], and sex [F > M; F(1,181)=11.5, *p*<0.001] on body temperature (Fig. 3A). In addition, a significant time-by-sex interaction effect [F(1,181)=3.7, *p*=0.05] and a nonsignificant trend for a treatment-by-time interaction effect [60 min >30 min; F(1,181)=3.7, *p*=0.05] on hypothermic response were detected. Females exhibited the largest response to treatment and the magnitude of the treatment effect on hypothermia was greatest at 60 min post-injection.

There was a significant main effect of sex on initial response to THC [F(1,28)=8.6, *p*<0.01] at 30 min post-injection. There were no main effects of strain or sex-by-strain interaction effects on initial response to the hypothermic effects of THC at this time point. At 60 min post-injection of THC, there were significant main effects of sex [F(1,89)=8.4, *p*<0.01] and strain [F(1,89)=4.0, *p*<0.05] on hypothermic initial response to THC. At both post-injection time points, females exhibited greater initial sensitivity to the hypothermic effects of THC relative to males (Fig. 3B). At the 60-min time point, the D2 strain exhibited greater initial sensitivity to the hypothermic effects of THC relative the B6 strain (Fig. 3B).

At 60 min post-injection (maximum initial response to THC), rapid tolerance to the hypothermic effects of THC upon second exposure accounted for a 50% or greater change in the direction of baseline body temperature (∼70% in B6 females, ∼50% in B6 males, ∼80% in D2 females, and ∼65% in D2 males; Fig. 3A). In contrast to the mobility trait, the return to baseline was complete by the third exposure to THC in both strains, sexes, and post-injection time points (Fig. 3A).

At 30 min post-injection of THC, there was a significant main effect of sex on rapid tolerance to THC-induced hypothermia [F(1,28)=12.8, *p*<0.05)]. At 60 min post-injection of THC, there was a significant main effect of both sex [F(1,81)=9.6, *p*<0.01)] and strain [F(1,81)=4.2, *p*<0.05)] on rapid tolerance to hypothermia. Relative to males, females exhibiting significantly greater rapid tolerance to the hypothermic effects of THC at both time points and the D2 strain exhibited greater rapid tolerance at 60 min post-injection relative to the B6 strain (Fig. 3C).

### Strain differences in the antinociceptive response to THC

**FIG. 4.**
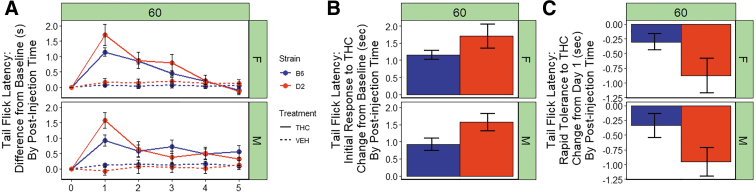
Antinociceptive response to THC. **(A)** Tail withdrawal latency in response to a thermal stimulus is shown for B6 and D2 females (top panel) and males (bottom panel) 60 min post-injection of THC (10 mg/kg) or VEH. Trait data for every individual have been transformed relative to baseline (day 0) by subtracting trait values for each day by the trait values for day 0 and averaging data by strain, sex, treatment, day, and post-injection time. Data represented as mean±SEM. A significant increase in tail flick latency following THC treatment is evident in both strains and sexes. Rapid tolerance upon second exposure is more prominent in the D strain and desensitization is nearly complete in both strains by the fifth treatment, especially in females. **(B)** Initial response to THC is shown as the difference between day 1 and 0 (baseline) for the THC treatment group (average of every individual's difference score). The D2 strain (both sexes combined) demonstrates significantly greater analgesia after a single exposure to THC relative to the B6 strain. **(C)** Rapid desensitization to THC is shown as the difference between day 2 and 1 (THC treatment group only). Significant rapid desensitization to the analgesic effects of THC upon second exposure was observed for the B6 strain relative to the D2 strain.

We observed significant main effects of treatment [THC > VEH; F(1,152)=50.6, *p*<0.001] and strain [D2 > B6; F(1,152)=9.8, *p*<0.01] on tail flick latency in response to a thermal stimulus (Fig. 4A). The D2 strain exhibited longer tail flick latencies relative to the B6 strain. There were no significant main effects of sex and there were no significant interaction effects on tail flick latency.

There was a significant strain difference in initial response to the antinociceptive effects of THC upon first exposure relative to baseline (Fig. 4B) with the D2 strain exhibiting greater sensitivity relative to the B6 strain [F(1,107)=6.5, *p*<0.05)]. No significant sex differences in initial response were observed.

For both strains and sexes, rapid tolerance to the antinociceptive effects of THC upon second exposure accounted for an ∼30%–60% change in the direction of baseline (Fig. 4A). D2 females and males exhibited greater rapid tolerance (∼50% and 60%, respectively) relative to B6 females and males (∼30% and 40%, respectively). The return to baseline was mostly complete in females of both strains by the fourth exposure to THC; however, males of both strains did not completely return to baseline by the fifth exposure (Fig. 4A). There was a significant main effect of strain [F(1,100)=7.4, *p*<0.01)] on rapid tolerance to the antinociceptive effects of THC, and D2 mice exhibited greater rapid tolerance to the analgesic effects of THC upon second exposure relative to B6 mice (Fig. 4C). No significant main effect of sex or sex-strain interaction effects on rapid tolerance were observed.

## Discussion

In this study, we provide the first comprehensive sex by strain comparison of initial response and rapid tolerance to THC, the main psychoactive component in recreational use cultivars of cannabis. We report strain differences in initial response to the ataxic (B6 > D2) and antinociceptive/analgesic effects (D2 > B6) of THC (10 mg/kg, *i.p.*). We also observe strain differences in rapid tolerance to the analgesic effects of THC (D2 > B6) and sex differences in hypothermic responses to THC (F > M).

The response traits measured in our study—ataxia, hypothermia, and antinociception—are all due to the direct action of THC at CB1R, evidenced by the fact that genetic or pharmacological deletion of CB1R renders mice insensitive to the ability of THC and other CB1R agonists^45–49^ to produce these effects. Based on cell type-specific deletion of CB1R, the cellular mediators of these response traits may be projection neurons in cortex and striatum.^55^ Our study provides strong support for the hypothesis that genetic variants between B6 and D2 in CB1R signaling pathways, potentially in discrete cortical and/or striatal neuronal populations, may mediate strain differences in initial response and/or rapid tolerance to THC.

It is highly likely that pre-existing genetic variation between B6 and D2 strains is the main cause of differential sensitivity to the ataxic and analgesic effects of THC upon first exposure. In support of this hypothesis, short-term selective breeding (three generations) for sensitivity or resistance to the initial motor depressant effects of THC in adolescent F2 mice derived from B6 and D2 demonstrated that this trait was indeed moderately heritable and controlled by segregating gene variants between B6 and D2.^56^

The precise genetic factors driving differences in THC initial response between B6 and D2 genetic backgrounds are unknown; however, our group has reported pre-existing differences in CB1R protein levels in striatum of B6 and D2.^57^ This difference in striatal CB1R levels (B6 > D2) has the potential to directly mediate initial response variation between strains to the motor depressant (B6 > D2), but not the analgesic effects of THC (D2 > B6). However, the relationship between gene variants, CB1R levels, and trait variation is not so straightforward.

Importantly, there are no variants of predicted high impact in the cognate cannabinoid receptor 1 gene (*Cnr1*), no concomitant change in transcript levels, and no evidence of genetic regulation of transcript expression.^57^ Thus, strain differences in striatal CB1R levels are likely the result of genetic variation at other, yet to be identified gene loci involved in regulation of CB1R levels, endogenous cannabinoid levels, and/or signaling pathways.

Tolerance is likely caused by CB1R downregulation (receptor internalization resulting in decreased levels of the receptor) and/or desensitization (modifications of the receptor or signal transduction pathway that decrease signaling).^58,59^ Relative to the very rapid tolerance observed for the hypothermic effects of THC (∼3 days males and females) and the antinociceptive effects of THC (∼5 days in females), we observed much less tolerance to the ataxic effects of THC in males and females of both strains.

The striatum is a key modulator of psychomotor responses to drugs of abuse and CB1R in striatal neurons has been shown to mediate the psychomotor effects of THC.^55^ Consistent with our findings of decreased tolerance to the motor effects of THC, other human and animal studies have also reported lower levels of CB1R following repeated cannabinoid exposure in the striatum relative to cortex and hippocampus.^50,58,60,61^

Rapid tolerance following two consecutive exposures to THC was evident for hypothermia and analgesia traits. For both traits, the sex and/or strain with the highest level of initial sensitivity also showed the highest level of rapid tolerance at matched time points post-injection of THC. This inverse relationship suggests the involvement of the same underlying genetic factors and regulatory pathways. Genetic correlation between traits in populations derived from B6 and D2 or quantification of CB1R levels at baseline and following consecutive THC treatments could be used in future to interrogate the relationship between initial response and rapid tolerance in more detail.

Identification of sex differences in response to cannabis use and abstinence in humans and cannabinoid response in pre-clinical models has been hindered by the failure to include an equal representation of female to male subjects in the vast majority of studies.^62,63^ Nevertheless, sex differences in response to cannabis or cannabinoids have been reported in some clinical and pre-clinical studies,^64–68^ suggesting that sex, along with genetic factors, could contribute to individual variation in risk of adverse side effects of cannabis use and risk of CUD.

In this study, we have evaluated a balanced number of male and female subjects and report large sex differences, primarily for hypothermic responses (both initial sensitivity and rapid tolerance) to THC. Sex differences in rapid tolerance to the hypothermic effects of THC have also been reported in rats. Female Wistar rats displayed more rapid tolerance to inhaled THC (200 mg/mL x2 daily) relative to males,^69^ as did female Sprague-Dawley rats (30 mg/kg THC delivered x2 daily *s.c.*) relative to males and ovariectomized females.^70^ Taken together, these studies suggest a possible role for estrogen, at least in the development of tolerance to the hypothermic effects of THC. However, the exact signaling mechanism and underlying circuitry influencing sex differences in response to THC have not been elucidated.

There are several caveats to our study. First, we were primarily focused on initial response and rapid tolerance to THC. Thus, our phenotyping pipeline explored an abbreviated exposure to THC spanning five daily exposures and does not address the effects of chronic cannabinoid exposure and withdrawal. Second, humans typically consume cannabis in the native state and are therefore exposed to ∼100 constituent cannabinoids whose targets and interactions are still not well understood. In this study, we have only profiled the response to a single main psychoactive component, THC. However, further investigation with cannabinoid mixtures is warranted and could help elucidate the molecular mechanisms underlying the response to other cannabinoids, such as CBD, or effects from the combined actions of constituent phytocannabinoids and terpenes.

In conclusion, our study provides strong evidence that genetic factors in the form of segregating variants between B6 and D2 genomes influence response to THC. Ultimately, forward genetic strategies in mouse populations with divergent behavioral and physiological responses to THC and other cannabis constituent components can be leveraged to identify genes that reduce adverse health consequences and/or maximize therapeutic properties of cannabis or cannabinoid-based therapeutics.

Genetic populations derived from B6 and D2, such as the recombinant inbred BXD population, can now be used to identify the enigmatic genetic factors modulating cannabinoid initial sensitivity and rapid tolerance. Derived by crossing the highly genetically and phenotypically divergent B6 and D2 strains followed by inbreeding of the resulting progeny, the BXD family is the largest and best characterized genetic reference population currently available.

This population is segregating for ∼6 million sequence variants, multiple levels of molecular and behavioral data have been quantified for many individual BXD strains, and this family has been used extensively to study genetic variation in traits related to cognitive function,^71–77^ anxiety,^40,78,79^ schizophrenia,^80^ and response to drugs of abuse such as ethanol, nicotine, methamphetamine, cocaine, and opioids.^39–43^ The availability of deep phenome data and marked trait variation in cognitive, sensorimotor, anxiety, and addiction-like behavior makes the BXDs an ideal population for investigating behavioral and physiological responses to THC and other cannabinoids. Future phenotyping efforts quantifying initial sensitivity and rapid tolerance to THC in the BXD panel are expected to result in the identification of key endocannabinoid signaling genes mediating the response to THC.

## Abbreviations Used

CB1R: cannabinoid receptor 1
CUD: cannabis use disorder
HSD: honest significant difference
THC: Δ9–tetrahydrocannabinol
VEH: vehicle
